# Supplementary material for: Pregnancy-associated plasma protein A in maternal serum for predicting early gestational diabetes mellitus: a systematic review and meta-analysis
Source: PeerJ. 2025 Sep 2;13:e19825. doi: 10.7717/peerj.19825 (PMC12422257; doi:10.7717/peerj.19825)
Supplement: Supplemental Information 2 [file peerj-13-19825-s002.docx]

**Table S1** **The search strategy (Pubmed)**

| Search number | Query | Results |
| --- | --- | --- |
| 1 | diabetes, gestational"[MeSH Terms] | 19236 |
| 2 | "diabetes mellitus gestational"[Title/Abstract] OR "gestational diabetes"[Title/Abstract] OR "gestational diabetes mellitus"[Title/Abstract] OR "maternal gestational diabetes mellitus"[Title/Abstract] OR "pregnancy diabetes"[Title/Abstract] OR "pregnancy diabetes mellitus"[Title/Abstract] OR "Pregnancy Induced Diabetes"[Title/Abstract] | 24275 |
| 3 | "pregnancy associated plasma protein a"[MeSH Terms] | 2101 |
| 4 | (#1 OR #2) AND #3"pregnancy associated plasma protein a"[Title/Abstract] OR "pregnancy associated plasma protein a"[Title/Abstract] OR "pregnancy associated alpha plasma protein"[Title/Abstract] OR "PAPP A"[Title/Abstract] OR "insulin like growth factor binding protein 4 protease"[Title/Abstract] OR "insulin like growth factor binding protein 4 protease"[Title/Abstract] OR "IGFBP 4 Protease"[Title/Abstract] OR "IGFBP 4 Metalloproteinase"[Title/Abstract] | 2531 |
| 5 | (#1OR #2) AND (#3 OR #4) | 1832 |
