# Supplementary material for: Pregnancy-associated plasma protein A in maternal serum for predicting early gestational diabetes mellitus: a systematic review and meta-analysis
Source: PeerJ. 2025 Sep 2;13:e19825. doi: 10.7717/peerj.19825 (PMC12422257; doi:10.7717/peerj.19825)
Supplement: Supplemental Information 3 [file peerj-13-19825-s003.docx]

## Systematic Review and/or Meta-Analysis Rationale

For systematic reviews / meta-analyses, authors need to provide the following information:

1.The rationale for conducting the systematic review / meta-analysis.

**Reply:**

We aim to integrate data to evaluate the utility of PAPP-A in the early diagnosis of GDM, thereby enhancing early detection and intervention strategies for GDM. This will provide evidence-supported guidance for clinical practice, offer evidence-based insights for healthcare professionals and public health policymakers, and identify potential avenues for future research.

**2.**The contribution that it makes to knowledge in light of previously published related reports, including other meta-analyses and systematic reviews.

**Reply:**

In recent years, with the publication of more relevant studies, the relationship between PAPP-A and GDM has been explored, but the results have not been consistent. For example, one study showed that PAPP-A has a high diagnostic efficacy for GDM (AUC: 0.82), while another study reached the opposite conclusion (AUC: 0.542). This study provides a more comprehensive and objective evaluation by synthesizing data from multiple studies. Through systematic review and Meta-analysis, this study comprehensively assessed the diagnostic efficacy of PAPP-A in detecting GDM risk among pregnant women in different geographical regions.
